# Supplementary figures and images for: Variation of soil bacterial communities along a chronosequence of Eucalyptus plantation
Source: PeerJ. 2018 Sep 24;6:e5648. doi: 10.7717/peerj.5648 (PMC6160830; doi:10.7717/peerj.5648)

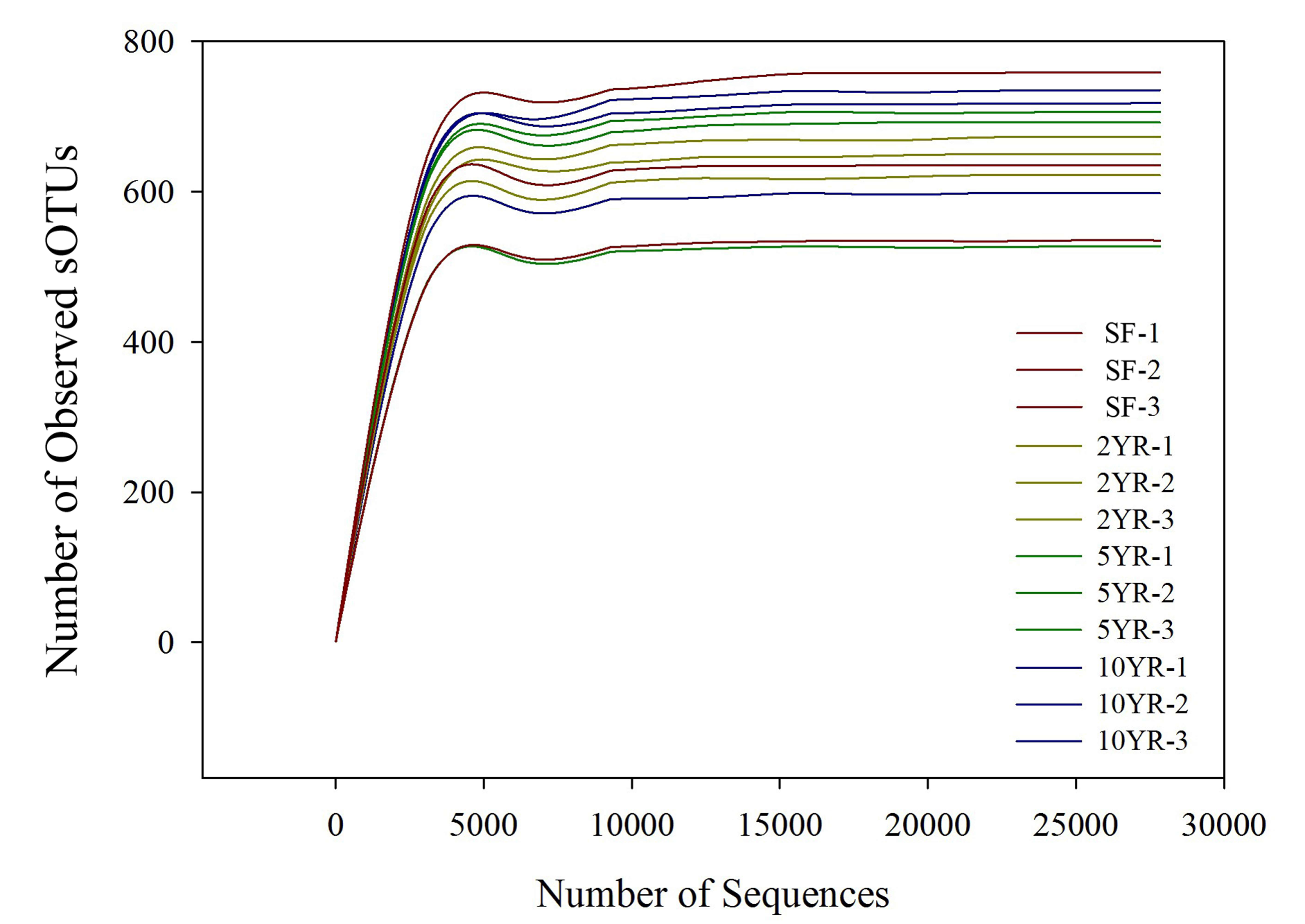

Supplement: Figure S1 [file peerj-06-5648-s001.png]

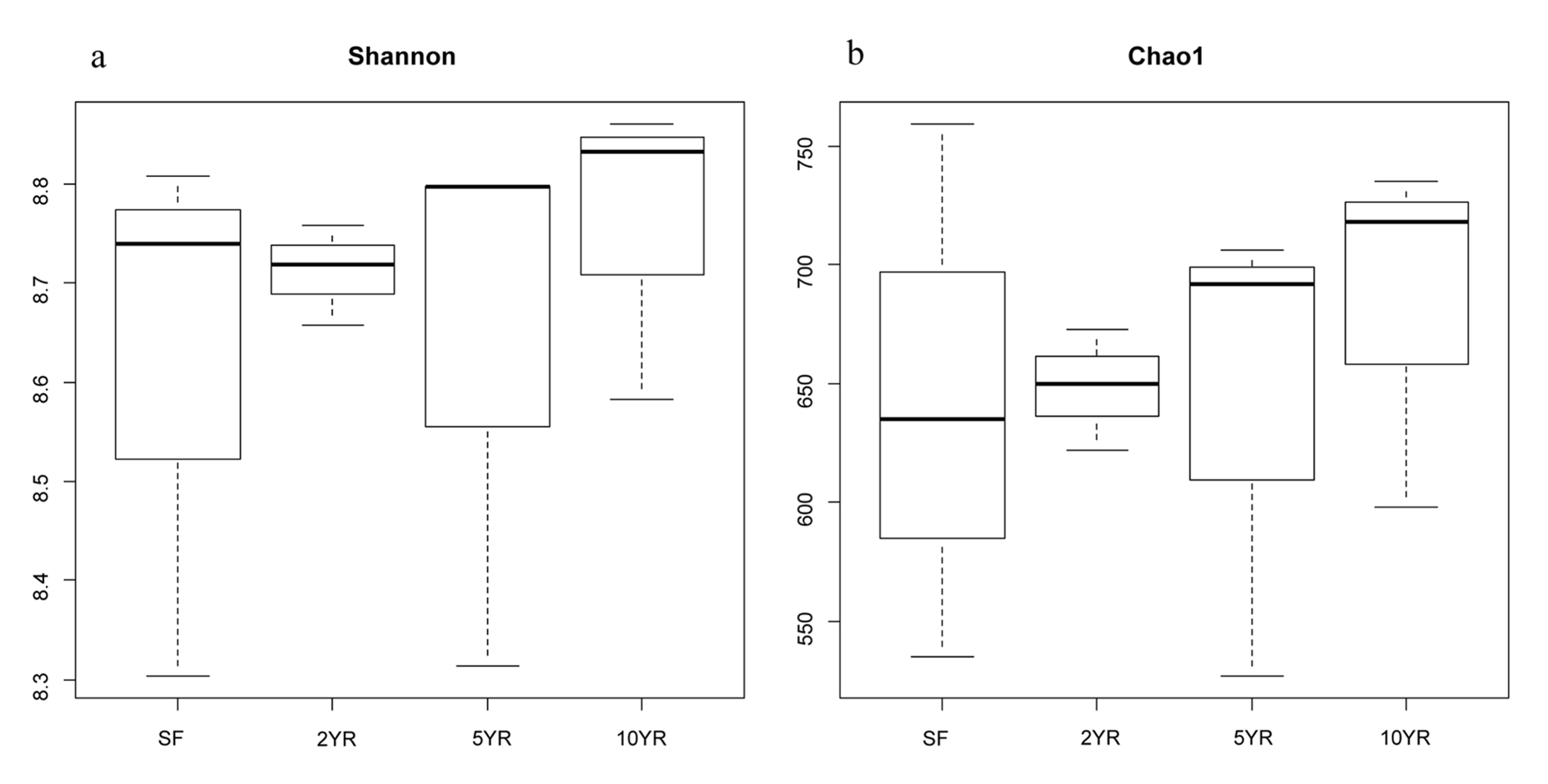

Supplement: Figure S2 [file peerj-06-5648-s002.png]

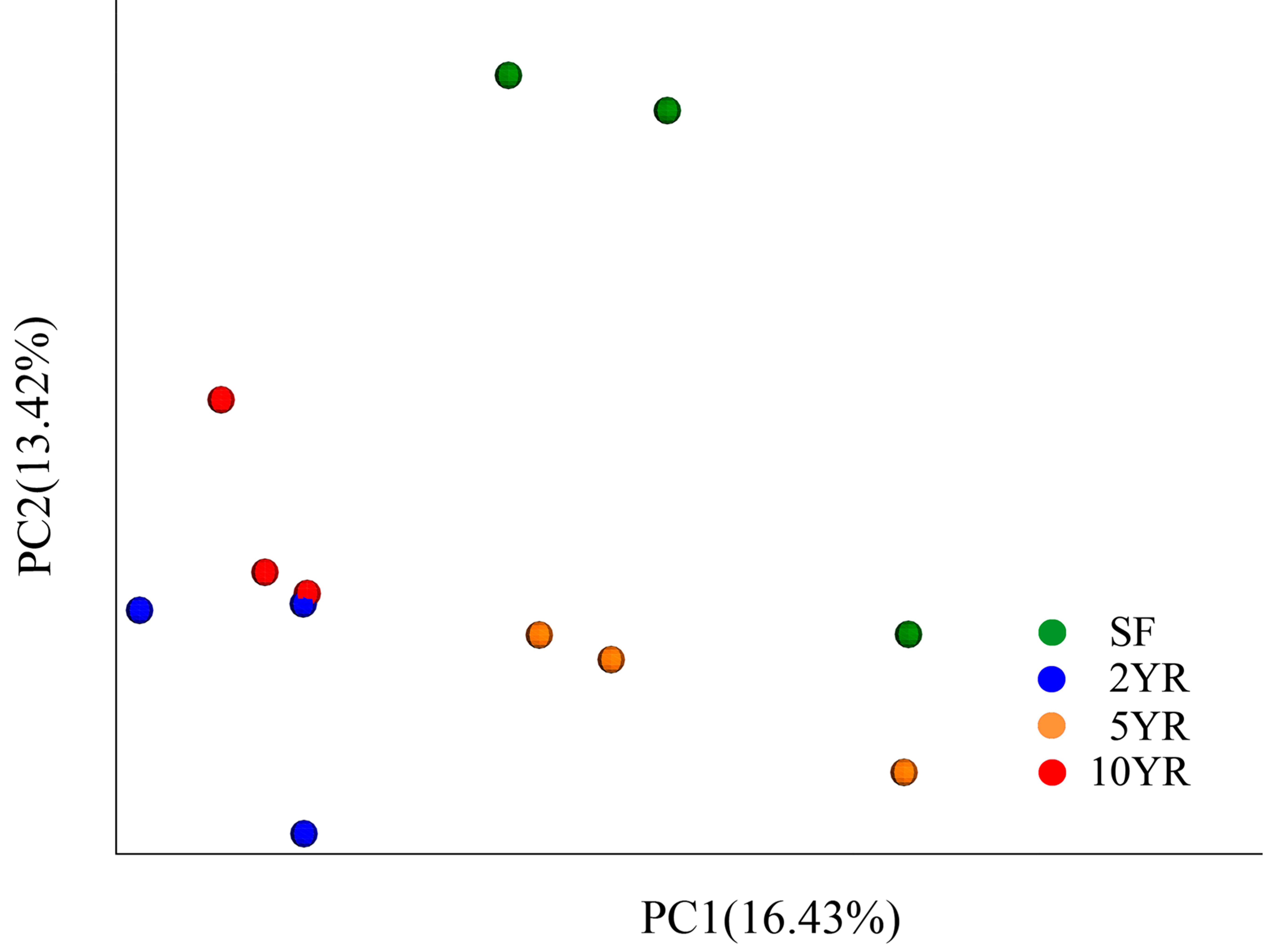

Supplement: Figure S3 [file peerj-06-5648-s003.png]

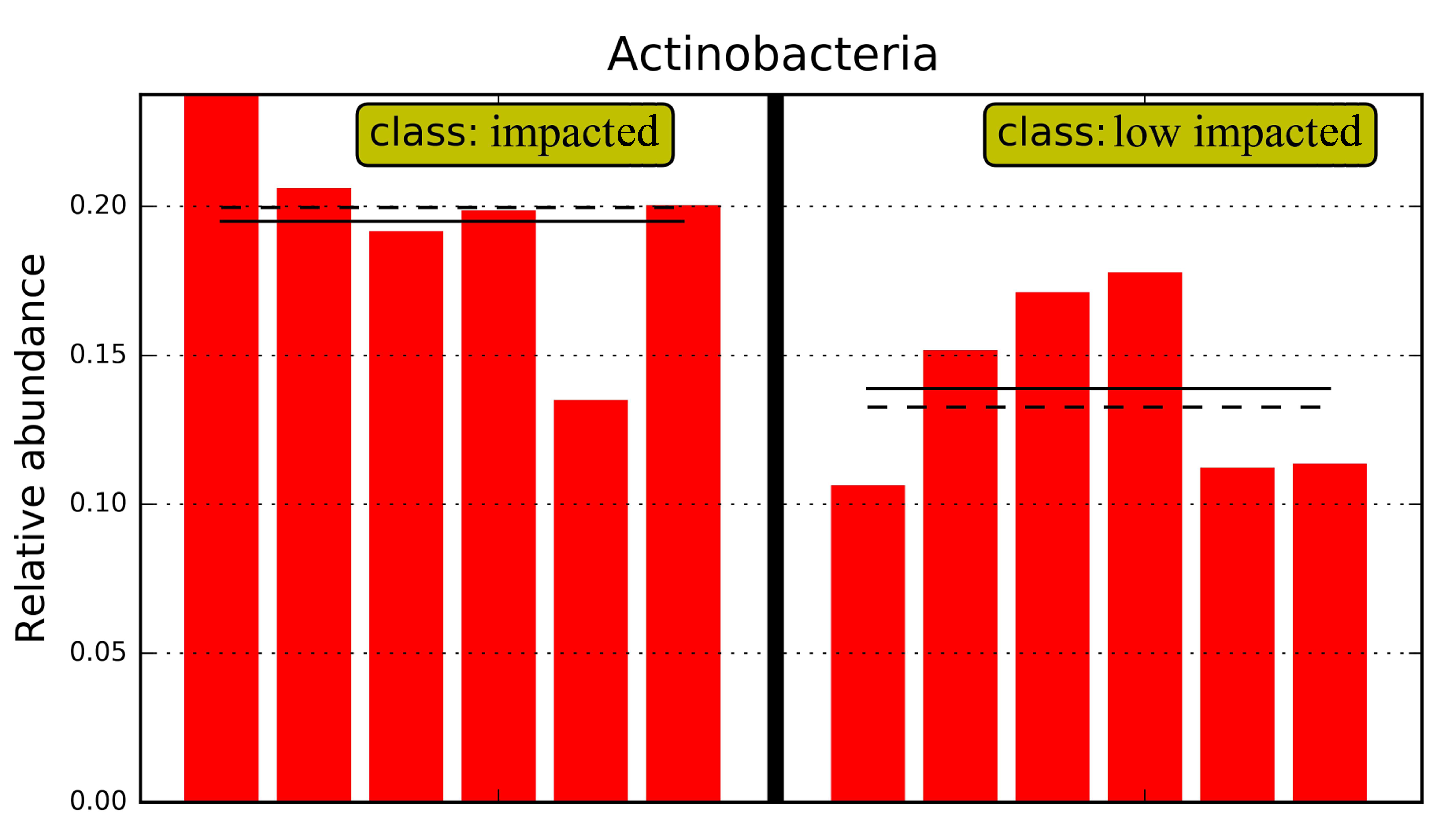

Supplement: Figure S5 [file peerj-06-5648-s005.png]

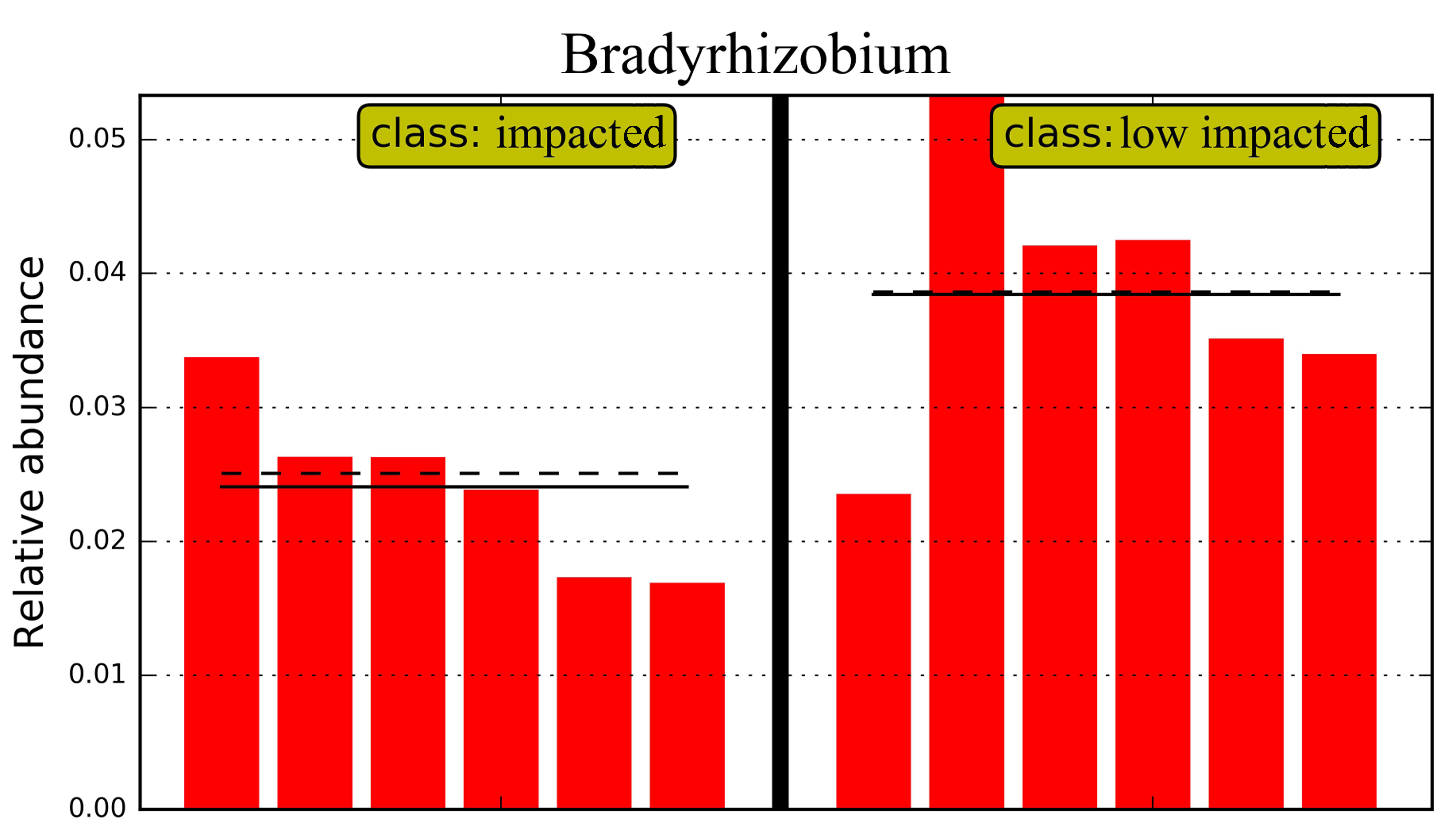

Supplement: Figure S6 [file peerj-06-5648-s006.png]

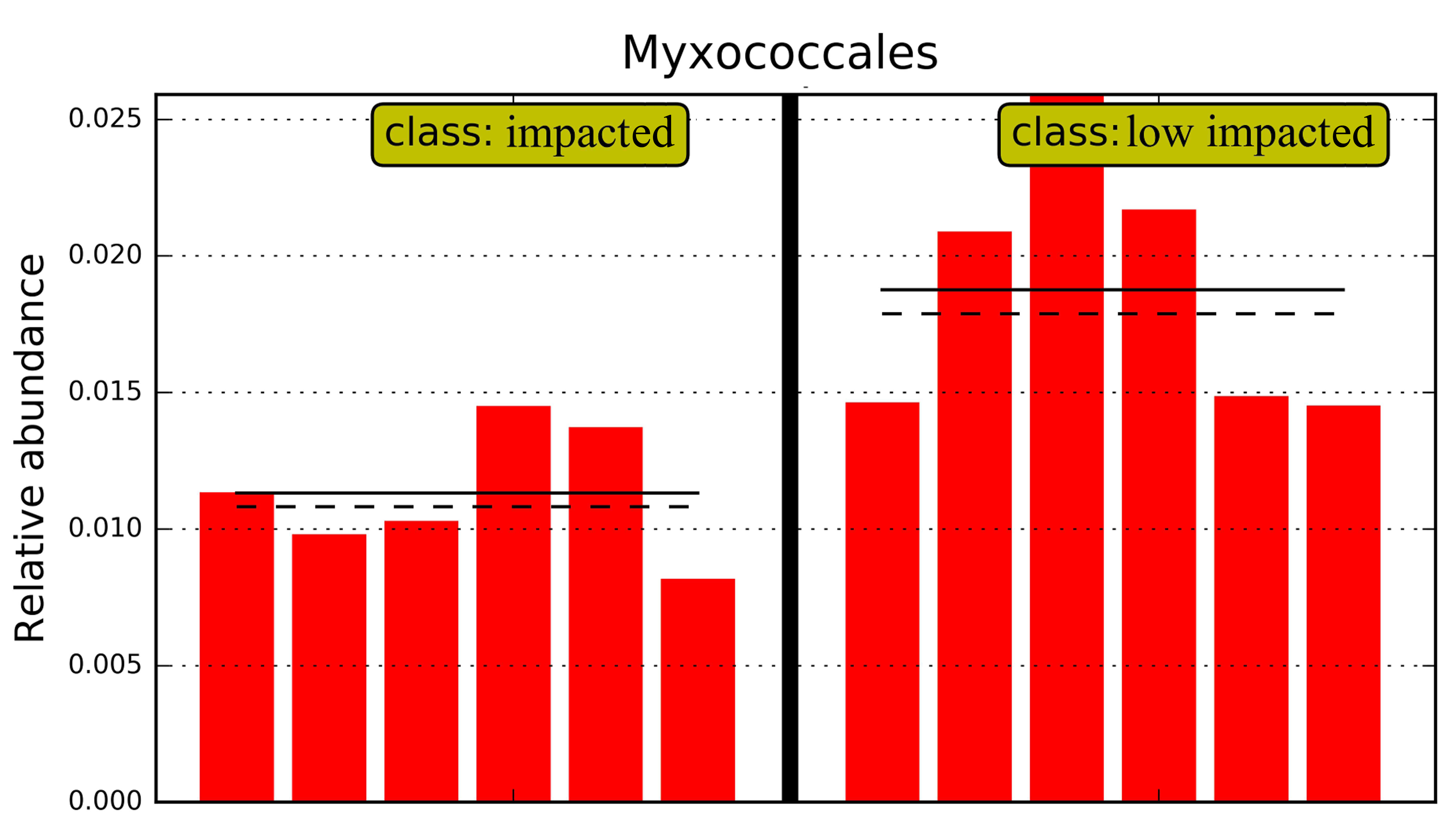

Supplement: Figure S7 [file peerj-06-5648-s007.png]
