# Supplementary material for: Variation of soil bacterial communities along a chronosequence of Eucalyptus plantation
Source: PeerJ. 2018 Sep 24;6:e5648. doi: 10.7717/peerj.5648 (PMC6160830; doi:10.7717/peerj.5648)
Supplement: Table S1 [file peerj-06-5648-s008.docx]

| Genus/Treatment | SF | 2YR | 5YR | 10YR |
| --- | --- | --- | --- | --- |
| *Rhodoplanes* | 7.48±1.99a | 6.74±0.99a | 7.24±1.77a | 8.10±1.15a |
| *norank_o__Actinomycetales* | 5.88±1.11a | 7.73±1.74a | 5.75±1.78a | 6.70±1.56a |
| *norank_f__Rhodospirillaceae* | 6.58±2.46a | 8.84±1.67a | 7.47±1.95a | 6.74±1.23a |
| *norank_o__Acidimicrobiales* | 2.61±1.11ab | 3.84±0.32a | 2.09±0.75b | 3.66±0.82a |
| *norank_p__WPS-2* | 2.10±1.03ab | 2.75±0.17a | 1.41±0.56b | 2.90±0.28a |
| *norank_o__Ellin329* | 1.73±0.21b | 2.60±0.26a | 1.76±0.17b | 2.47±0.55a |
| *norank_f__Conexibacteraceae* | 1.22±0.46bc | 2.59±0.26a | 0.99±0.28c | 1.83±0.43b |
| *norank_f__Acetobacteraceae* | 3.21±1.1a | 2.41±0.04ab | 1.67±0.5b | 1.99±0.31b |
| *Bradyrhizobium* | 3.78±1.43a | 2.76±0.42ab | 3.56±0.42a | 1.86±0.38b |
| *norank_f__Caulobacteraceae* | 1.39±0.09a | 1.55±0.11a | 1.04±0.16b | 1.46±0.2a |
| *norank_o__Actinomycetales* | 0.89±0.35b | 1.56±0.25a | 0.62±0.04b | 1.09±0.43ab |
| *norank_f__Acetobacteraceae* | 1.04±0.18a | 1.05±0.15a | 1.05±0.16b | 1.05±0.17ab |
| *Candidatus Koribacter* | 0.81±0.4b | 1.50±0.15ab | 1.11±0.76ab | 1.94±0.54a |
| *norank_o__iii1-15* | 0.65±0.37b | 0.65±0.05b | 1.80±1.04a | 1.41±0.14ab |
| *norank_f__Methylocystaceae* | 1.61±0.42a | 1.02±0.39b | 0.79±0.14b | 0.96±0.16b |
| *norank_o__Myxococcales* | 1.95±0.53a | 1.00±0.08b | 1.63±0.38ab | 1.17±0.33b |
| *norank_c__JG37-AG-4* | 0.23±0.18c | 1.06±0.36a | 0.47±0.07bc | 0.78±0.09ab |
| *Mycobacterium* | 0.95±0.28ab | 1.24±0.16a | 1.06±0.34ab | 0.72±0.03b |
| *norank_o__Solirubrobacterales* | 0.16±0.14c | 0.89±0.14a | 0.08±0.05c | 0.62±0.17b |
| *norank_c__TK17* | 0.36±0.06ab | 0.35±0.06ab | 0.25±0.05b | 0.43±0.09a |
| *Conexibacter* | 0.31±0.08ab | 0.48±0.1a | 0.14±0.08b | 0.29±0.25ab |
| *Skermanella* | 1.21±1.03a | 0.68±0.13ab | 0.13±0.1b | 0.53±0.38ab |
| *Azospirillum* | 0.53±0.1b | 0.98±0.11a | 0.82±0.39ab | 0.43±0.08b |
| *norank_f__Pseudonocardiaceae* | 0.02±0.04b | 0.12±0.12b | 0.04±0.04b | 0.38±0.09a |
| *norank_c__TM7-1* | 0.35±0.09b | 0.59±0.16a | 0.29±0.06b | 0.46±0.15ab |
