# Supplementary material for: Variation of soil bacterial communities along a chronosequence of Eucalyptus plantation
Source: PeerJ. 2018 Sep 24;6:e5648. doi: 10.7717/peerj.5648 (PMC6160830; doi:10.7717/peerj.5648)
Supplement: Table S3 [file peerj-06-5648-s010.docx]

| Samples | pH | SOC | TN | TP | NH4+ | NO3- |
| --- | --- | --- | --- | --- | --- | --- |
|  |  | (g/kg) | (g/kg) | (g/kg) | (mg/kg) | (mg/kg) |
| Control | 4.28±0.04a | 17.85±0.61c | 0.77±0.02b | 0.17±0.00b | 5.34±0.68a | 4.06±0.64a |
| Three months | 4.23±0.03a | 21.72±3.21b | 0.90±0.11b | 0.19±0.01b | 9.46±4.52a | 4.27±3.67a |
| Six months | 4.36±0.31a | 65.66±0.35a | 2.14±0.09a | 0.26±0.03a | 6.52±1.91a | 3.66±0.52a |
